# Biology of the RANKL–RANK–OPG System in Immunity, Bone, and Beyond

**DOI:** 10.3389/fimmu.2014.00511

**Published:** 2014-10-20

**Authors:** Matthew C. Walsh, Yongwon Choi

**Affiliations:** ^1^Department of Pathology and Laboratory Medicine, University of Pennsylvania Perelman School of Medicine, Philadelphia, PA, USA

**Keywords:** osteoimmunology, TRAF6, TRANCE, RANKL, TNFSF11, TNFRSF11, mTECs, rheumatoid arthritis

## Abstract

Discovery and characterization of the cytokine receptor-cytokine-decoy receptor triad formed by receptor activator of nuclear factor kappa-B ligand (RANKL)–receptor activator of NF-κB (RANK)–osteoprotegerin (OPG) have led not only to immense advances in understanding the biology of bone homeostasis, but have also crystalized appreciation of the critical regulatory relationship that exists between bone and immunity, resulting in the emergence of the burgeoning field of osteoimmunology. RANKL–RANK–OPG are members of the tumor necrosis factor (TNF) and TNF receptor superfamilies, and share signaling characteristics common to many members of each. Developmentally regulated and cell-type specific expression patterns of each of these factors have revealed key regulatory functions for RANKL–RANK–OPG in bone homeostasis, organogenesis, immune tolerance, and cancer. Successful efforts at designing and developing therapeutic agents targeting RANKL–RANK–OPG have been undertaken for osteoporosis, and additional efforts are underway for other conditions. In this review, we will summarize the basic biology of the RANKL–RANK–OPG system, relate its cell-type specific functions to system-wide mechanisms of development and homeostasis, and highlight emerging areas of interest for this cytokine group.

## Introduction

Functional diversity typifies the cytokines and receptors of the tumor necrosis factor (TNF) and tumor necrosis factor receptor (TNFR) superfamilies. TNF superfamily (TNFSF) members are broadly expressed in a variety of tissues and organ systems, and are commonly associated with expression on cells of the immune system. In recent years, studies of TNF/TNFR superfamily members have been responsible for elucidating previously unrecognized linkages between the immune system and other biological systems, as well as previously unrealized networks controlling various disease conditions (1, 2). An important example is the system consisting of the cytokine receptor activator of nuclear factor kappa-B ligand [RANKL; additionally identified as TNF-related activation-induced cytokine (TRANCE), osteoclast differentiation factor (ODF), and TNFSF11], its signaling receptor receptor activator of NF-κB (RANK), and the soluble decoy receptor osteoprotegerin (OPG). The RANKL–RANK–OPG system was originally discovered through parallel efforts in the late 1990s that identified it as important to immunity, primarily via actions on dendritic cells (DCs) (3, 4), and as important to bone homeostasis through regulation of osteoclasts (OCs) (5, 6). Work employing various genetically deficient mouse models has shown the critical role of the RANKL–RANK–OPG system plays in bone and immunity – significantly contributing to the emergence of the field of osteoimmunology – as well as organogenesis, and disease conditions including cancer and rheumatoid arthritis (RA). In this review, we will provide a summary of current understanding of the biological functions of the RANKL–RANK–OPG system in development, homeostasis, immunity, and disease, as well as ongoing efforts to target RANKL–RANK–OPG to prevent or fight diseases like osteoporosis and cancer.

## Receptor Activator of Nuclear Factor kappa-B Ligand

Receptor activator of nuclear factor kappa-B ligand (3), which was independently discovered by four groups, is alternatively named TRANCE (4), ODF (6), osteoprotegerin ligand (OPGL) (5), and designated TNFSF11. The gene encoding the RANKL protein, *Tnfsf11*, is located on human chromosome 13q14, and a conserved syntenic region on mouse chromosome 14 (4). RANKL protein is a type II membrane protein bearing close homology to TNFSF members TRAIL, FasL, and TNF-a (4). Full-length murine RANKL is 316 amino acids, shares 83% sequence homology with human RANKL (4), and consists of a C-terminal extra-cellular receptor-interacting domain and a transmembrane domain, but is found in both membrane-bound and soluble forms (7). Cleavage of soluble RANKL from the membrane-embedded portion is mediated by the metalloprotease–disintegrin TNF-a convertase (TACE) (8, 9). Further, three distinct isoforms of RANKL message have been identified, the shortest of which – lacking the intracellular and transmembrane domains – may have inhibitory function (10). These data comport with observations that soluble RANKL is less efficient at mediating osteoclastogenesis (11). RANKL expression has been detected in various tissues including T lymphocytes (4), osteoblasts (OBs), osteocytes and bone stroma, and lung (5, 6, 12, 13). Throughout development RANKL mRNA can be detected in the brain, heart, kidneys, skeletal muscle, and skin of mouse embryos, and has been specifically identified in E15 chondrocytes (5, 12). Expression of RANKL is highly inducible, and is regulated by various osteoactive factors including glucocorticoids (14), Vitamin D3 [1,25(OH)2D3] (6, 15), IL-1 (16), TNF-a (16), TGF-b (17), Wnt ligands (18), and LPS (19). Binding studies show that RANKL can bind to both the functional receptor RANK and the decoy receptor OPG (3, 5).

## Receptor Activator of NF-κB

**Figure 1:**
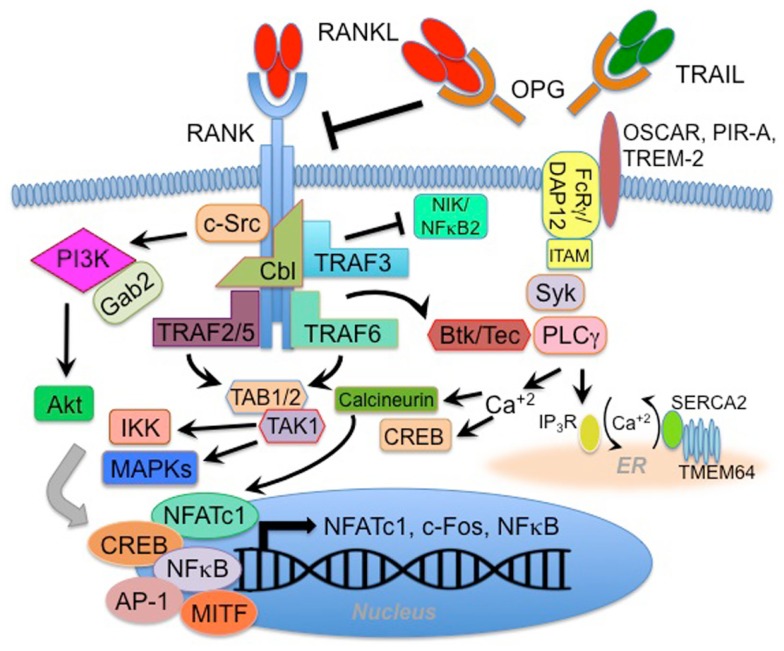
**RANK signaling pathways**. The RANK receptor lacks intrinsic enzymatic activity and therefore utilizes interaction with adaptor and docking proteins, including TRAFs 2, 3, 5, and 6, Gab2, and Cbl to activate downstream signaling. Gab2 and Cbl are associated with RANK-mediated activation of c-Src, PI3 kinase (PI3K), and Akt, while TRAFs 2 and 6 can activate the TAB1/TAB2/TAK1 complex, which (along with other upstream kinases) leads to activation of IKKβ and MAP kinases (MAPK). Activation of these pathways promotes translocation and activation of transcription factors including NFATc1, CREB, NFκB, AP-1, and MITF. Specific RANK-activated gene transcription varies depending on cell-type, but often involves feed forward expression of NFATc1, c-fos, and NFκB-related genes. RANK-associated TRAF3 has been implicated in negative regulation of the non-canonical NFκB2 pathway through regulation of the upstream kinase NIK. Inhibition of NIK is mediated by the TRAF3 RING finger domain, and is overcome when RANK activation by RANKL triggers autophagic/lysosomal degradation of TRAF3. While many of these mechanisms may be generalizable to various RANK-expressing cell-types, some mechanisms appear thus far to be osteoclast (OC) lineage-specific. The best characterized of these OC-associated mechanisms involves synergistic signaling between RANK and ITAM motif-containing proteins DAP12 and FcRγ (which associate with cell surface receptors OSCAR, PIR-A, or TREM-2) to activate the Syk-PLCγ pathway and flux calcium. This activity enhances NFATc1 and CREB activities. Synergy with RANK occurs via coordinate activation of Btk/Tec. RANK further regulates calcium flux in OC lineage cells by a mechanism involving transmembrane protein 64 (TMEM64) interaction with the sarcoplasmic endoplasmic reticulum Ca(2+) ATPase 2 (SERCA2). This mechanism further promotes CREB and NFATc1 activity.

Receptor activator of NF-κB [(3); alternatively identified as TNF-related activation-induced cytokine receptor (TRANCE-R) (20) or osteoclast differentiation and activation receptor (ODAR) (21)] is the signaling receptor for RANKL. RANK has been designated TNFRSF11A, and is a type I 616 amino acid homo-trimerizing transmembrane protein containing four extra-cellular cysteine-rich pseudorepeats. Trimerization is promoted by interaction with RANKL (22). The human gene that encodes RANK, *Tnfrsf11a*, is located on chromosome 18q22.1 (3), and RANK message is detected in thymus, liver, colon, mammary glands, prostate, pancreas, bone marrow, heart, lung, brain, skeletal muscle, kidney, liver, and skin (3, 7, 23). RANK is strongly induced, especially on OC precursor cells, by M-CSF (24). Typical of TNFRSF members, RANK lacks intrinsic kinase activity and must rely on recruiting factors capable of activating downstream signaling pathways (summarized in Figure 1). As such, RANK intracellular signal transduction is mediated first through direct interaction with tumor necrosis factor receptor-associated factors (TRAFs), which are recruited upon receptor activation (25, 26). RANK interacts with TRAFs 1, 2, 3, 5 in a membrane-distal region of the 383 amino acid cytoplasmic tail, and with TRAF6 at a distinct membrane-proximal Pro-X-Glu-X-X-(aromatic/acid residue) binding motif (25–27). TRAF6 is critical activating mitogen-activated protein kinases (MAPKs) p38 and JNK, as well as the canonical NF-κB pathway in response to RANK signaling (8, 28, 29). TRAF6 utilizes the adapter TAB2 to interact with the MAPK kinase TAK1 to mediate RANK signaling (30). While RANK binds similar TRAFs as the related receptor CD40, it has been shown that stronger activation of TRAF6 may account for unique RANK function (31). Other interacting factors that may modulate RANK signaling include Grb2-associated binding protein 2 (Gab2) (32), epidermal growth factor receptor (EGFR) (33), four-and-a-half LIM domain 2 (FHL2) (34), Lyn (35), CYLD de-ubiquitinase (36), and TRAF family member-associated NF-κB activator (TANK) (37). It has been shown that RANK signaling can regulate calcium oscillation through downstream activation of regulator of G-protein signaling 10 (RGS10) (38), and that RANK-mediated calcium flux is itself regulated by transmembrane protein 64 (TMEM64) interaction with sarcoplasmic endoplasmic reticulum Ca(2+) ATPase 2 (SERCA2) (39). RANK also activates Src family kinase signaling in a manner that leads to Akt/PKB activation through interactions between TRAF6 and Cbl scaffolding proteins (40, 41). TRAF6-dependent RANK signaling has been shown to be negatively regulated via cross-talk with the IFN-g signaling pathway, employing a mechanism leading to TRAF6 ubiquitination and degradation (42). More recently, it has been shown that TRAF3 plays a key role in negatively regulating RANK-mediated activation of the non-canonical NF-κB pathway (43). One of the signaling properties of RANK that may distinguish it functionally from some other TNFR superfamily members is its capacity to activate both the canonical and non-canonical NF-κB pathways. At the level of gene regulation, RANK signaling is crucial for induction of the transcription factors c-fos and NFATc1/NFAT2 (44–46). The functional effects of RANK signal transduction are discussed further below.

## Osteoprotegerin

Osteoprotegerin, now designated as TNFRSF11B, was first identified through a discovery effort targeting TNFRSF homologs in rat, and human and mouse OPG homologs subsequently identified exhibited >85% homology (47). Independently discovered and alternatively named osteoclastogenesis inhibitory factor (OCIF) (48), TR1 (49), and follicular DC-derived receptor-1 (FDCR-1) (50) were found to be identical to OPG. OPG mRNA is detected in the calavaria, skin, liver, lung, and heart of the adult mouse, and peaks at days 7 and 15 in fetal tissue (47). OPG is expressed primarily by bone marrow stromal cells, but can be induced in B lymphocytes, DCs, and follicular DCs (50). OPG expression regulated both positively (e.g., TGF-b, IL-1, TNF, estrogen, and Wnt ligands) and negatively (e.g., prostaglandin E2 (PGE2) and glucocorticoids) by a wide array of factors, most of which are associated with bone homeostasis (51). Full-length OPG protein is 401 amino acids long, which is signal peptidase-cleaved to a 380 amino acid form containing four cysteine-rich N-terminal domains (domains 1–4), two death domain homologous regions (domains 5 and 6), and a C-terminal heparin-binding domain (domain 7) (52, 53), and then N-linked glycosylated, and secreted as a disulfide-linked homodimer (47–49). As such, OPG is believed to function primarily as a decoy receptor, modulating interactions between ligands and signaling receptors. The high-affinity binding partner for OPG appears to be RANKL, but it has also been shown to bind with low affinity (3.0 nM at 4°C and 400 nM at 37°C) to the TNFSF member and pro-apoptotic factor, TRAIL (54). *In vitro* and pre-clinical studies suggest that OPG–TRAIL interactions may be relevant to apoptosis of tumor cells (52). Additional potentially physiologically relevant ligands of OPG include syndecan-1, glycosaminoglycans (GAGs), von Willebrand Factor, and Factor VIII von Willebrand Factor complex (52).

## Organogenesis

Receptor activator of nuclear factor kappa-B ligand–receptor activator of NF-κB has emerged as a critical signaling pathway for the cellular differentiation and development of epithelial tissues in various organs. These RANK-dependent processes have important implications not only for development, but also for regulating immunity and cancer.

### Lymph nodes

Characterization of RANKL- and RANK-deficient mice revealed failed or abnormal development of secondary lymphoid tissues, including lymph nodes, Peyer’s patches, cryptopatches, and spleen (55–58). During embryogenesis, secondary lymphoid tissue development occurs as RANKL signals through RANK on lymphoid tissue inducer (LTi) cells recruited to a rudimentary anlage composed of lymphoid tissue organizer (LTo) progenitor cells. LTi cells then transmit lymphotoxin a/b (LTa/b) to LTo progenitors that express LTbR, which drives development of mature LTo cells. This event triggers a feedback loop of RANKL and RANK expression by mature LTo cells, which amplifies LTi growth and tissue organization (59). The relevant RANK signaling in this context appears to be mediated by the transcriptional regulator Id2 (60).

### Mammary glands

Receptor activator of nuclear factor kappa-B ligand- and RANK-deficient mice also exhibit defective mammary gland development (61, 62), which requires RANK-triggered activation of the IKK-alpha kinase domain in order to activate the non-canonical NF-κB pathway (63). RANK signaling in the context of mammary gland development is also associated with expression of cyclin D1, Id2, and Id4 (59). It has recently been shown that RANK signaling is required for mammary epithelial stem cell activation (64, 65). Further, RANK signaling has been shown to be required for progesterone-driven proliferation of normal breast tissue (66).

### Medullary thymic epithelial cells

A critical role for RANKL–RANK signaling has been established in thymic organ development, and specifically for the epithelial lineage cells required for negative selection of developing T cells (67, 68). The thymus educates self-tolerant T cells by eliminating those expressing potentially self-reactive TCRs and by generating the immunosuppressive T cells that are essential for preventing autoimmune disease. Epithelial cells localized in the thymic medulla called medullary thymic epithelial cells (mTECs) are non-hematopoietic in origin and are essential for negative selection (68). Recent studies have revealed that mTECs also contribute to the selection and survival of immunosuppressive Foxp3-positive regulatory T cells (Tregs) (68, 69). The significant decrease in mTECs in the absence of RANKL provision by thymocytes suggests that mTEC differentiation is actually driven by the cells that mature mTECs subject to negative selection (68). In fact, this process appears to be initiated in the neo-natal period by innate RANKL-expressing gamma-delta lineage cells before the appearance of alpha-beta cells in the thymus (70). mTECs require signaling through each of the TNFRSF members LTbR, CD40, and RANK in order to induce sufficient expression of the critical factors autoimmune regulator (Aire) and tissue-specific antigens (TSAs), but it had been unclear whether downstream activation by those receptors was qualitatively or quantitatively distinct. Cell-specific deletion of the non-canonical NF-κB inhibitor TRAF3 suggests that LTbR and CD40 are similar in their provision of non-canonical NF-κB activation, but that RANK apparently provides additional requisite signals during mTEC development (71). From genetic experiments, however, it is known that the relevant RANK signals are transduced by TRAF6 (67). Together, these data show that RANK-mediated signaling is required for developmental processes that critically affect immune regulation and homeostasis.

### M cells

Microfold cells are specialized intestinal epithelial-derived cells that make up roughly 10% of the follicle-associated epithelial (FAE) cells covering the gut-associated lymphoid tissues (GALT). Microfold (M) cells exhibit some morphological distinctions compared to neighboring enterocytes, but are most important for their enhanced capacity for phagocytosis and transcytosis of macromolecules, particulate antigens, and micro-organisms residing in the gut lumen (9). M cells are essential for transfer of antigens from orally acquired pathogens, such as Salmonella Typhimurium, to antigen-presenting cells, and subsequently, for optimal immune responses (9). Because of their importance to mucosal immunity, various efforts have aimed at determining the mechanisms of differentiation of M cells. Within the villous crypts of the FAE reside leucine-rich repeat-containing G-protein-coupled receptor-(Lgr5) expressing epithelial stem cells that are capable of differentiating into the various sub-lineage found in the gut, including enterocytes, goblet cells, enteroendocrine cells, tuft cells, Paneth cells, and M cells (9). Signals received depending on micro-anatomic location and neighboring cells can determine lineage specificity. It has been shown that stromal cells in the subepithelial dome of the FAE provide RANKL to RANK-expressing stem cells to induce M cell differentiation, and that in RANKL-deficient mice, M cells fail to develop unless sufficient exogenous RANKL is provided (57, 72). This data demonstrated an absolute requirement for RANK signaling during M cell development. This work further suggested that RANK signaling on M cell precursors is triggered by membrane-bound RANKL on stromal cells, such that cell–cell contact and FAE micro-architecture may be critical to M cell differentiation (57). RANKL has been shown to specifically induce the markers annexin A5 and myristoylated alanine-rich C-kinase substrate)-like protein 1 (MARCKS) on M cell precursor stem cells, but their significance in this context remains unclear (9, 73, 74). RANK signaling apparently acts in a critical manner to upregulate the ETS (E26 transformation-specific) transcription factor Spi-B, which was shown to be required for M cell maturation, and is responsible for cell-intrinsic expression of genes including glycoprotein 2 (Gp2), TNF alpha-induced protein 2 (Tnfaip2), and C–C motif chemokine ligand 9 (Ccl9) (73, 74). Finally, it remains to be determined why only a small proportion of Lgr5+ stem cells in the subepithelial dome of the FAE develop into stem cells despite ample exposure to stromal RANKL (9, 57). A possibility recently put forward might involve cross-talk between the RANK and Notch signaling pathways during M cell development, such that availability of given Notch ligands to M cell precursors determines whether RANK signaling is sufficient for M cell differentiation to proceed (9, 75).

## Bone Homeostasis

Bone is essential for providing skeletal strength, vital organ protection, a mineral reservoir for calcium, and a site for immune cell development. Bone cell homeostasis is maintained by the balanced functions of primarily two cell-types: OBs, which build bone, and OCs, which resorb bone (76, 77). In a continuous cycle, OCs remove bone by sealing off sections and secreting digestive acid into the lacunae, followed by OBs filling the cavity with new bone (76, 77). This constant remodeling of the bone matrix is necessary to maintain both skeletal strength and a reservoir for hematopoiesis. There is intense interest in understanding the mechanisms governing OC development and function. While OBs are mesenchymal lineage cells, OCs arise from myeloid hematopoietic precursor cells (76, 77). Interestingly, new OCs are generated largely through cell contact-dependent interactions between OC precursors and OBs in the bone stroma (76, 77). RANKL–RANK signaling was shown early on to be a potent driver of OC differentiation even under minimal *in vitro* conditions (5). OC precursors require the growth factor M-CSF for OC precursor growth and RANK induction (78, 79), and then the RANK-induced transcription factors c-fos (80), NFATc1/NFAT2 (44, 46), and NF-κB members p50 and p52 (81, 82) [which also activated c-fos and NFATc1/NFAT2 in pre-OCs (83)] for OC development. Mice genetically deficient for any of these factors exhibit osteopetrosis, a thickening of the bones, due to reduced OC numbers and/or activity. RANKL–RANK-mediated activation of these pathways and induction of c-fos, NFATc1/NFAT2, and canonical and non-canonical NF-κB pathways during OC development is necessary for activation of OC-critical genes, including tartrate-resistant acid (TRAP), cathepsin-K, calcitonin receptor, as well as c-myc, to promote OC proliferation (76). RANKL–RANK signaling also plays a crucial role in the bone-resorbing function of mature OCs, which after αVβ3-mediated attachment of OCs to bone surfaces, leads to activation of Src-dependent pathways, including Syk, through complex formation involving RANK, Slp-76, Vav3, and Rac (76). Activation of these pathways and cytoskeletal rearrangements promote ruffled border formation via fusions between lysosomal secretory vesicles and the cytoplasmic membrane (76, 84). RANKL-deficient mice are severely osteopetrotic due to a cell non-autonomous defect in OC development, and additionally exhibit failed tooth eruption (common in developmental osteopetrosis), and diversion of hematopoiesis to the spleen and liver due to failed bone marrow cavity formation (58). Therefore, new bone formation occurs in the absence of RANK signaling, including intramembranous and endochondral ossification processes during fetal development, but bone remodeling is severely diminished. Further, RANK- and RANKL-deficient mice are exact phenocopies with respect to OC development, suggesting an exclusive relationship between the RANK–RANKL receptor–ligand pair (55). The critical source(s) of RANKL during normal bone remodeling is still debated, but evidence suggests that the source changes over development, such that hypertrophic chondrocytes provide RANKL for removal of trabecule during endochondral ossification, and trabecular osteocytes provide most RANKL for both mature bone remodeling and in response to mechanical stress (85, 86). Additionally, many well known osteotropic factors, including IL-1, IL-6, and IL-11 are believed to exert osteoclastogenic activity simply by inducing RANKL expression on OBs (78). The cytokine TNF-a promotes osteoclastogenesis, not only via direct stimulation of OC precursors (87), but also by inducing RANKL expression on stromal cells and RANK expression on OC precursor cells (88). TRAF6-deficient mice also exhibit severe osteopetrosis, confirming the critical relationship between this signaling adapter and RANK signaling (28, 29). Another study examining TRAF binding site in the RANK receptor suggests that TRAF6 might be most important for OC function and normal F-actin ring formation, and implying that signals through other TRAFs may also make important contributions to RANK-mediated osteoclastogenesis (89). However, a study using TRAF2-deficient OC precursors showed that while TRAF2 made a minor contribution to RANK signaling, it is primarily required for TNF-a-dependent osteoclastogenesis (90). Downstream of TRAF6, RANK signaling in OCs has been shown to activate JNK1 (91), Akt/PKB (41), p44/42 ERK (41), p38 MAPK (92), and the canonical NF-κB pathway (26). RANK-mediated activation of the c-src pathway links it to a critical requirement c-src for normal OC development (40, 41, 93, 94). Studies of OC differentiation have also identified a critical costimulatory pathway for RANK signaling involving immunoreceptor tyrosine based activation motif (ITAM)-containing receptors DNAX activation protein of 12 kDa (DAP12) and Fc-receptor g subunit (FcRg) (95). This costimulatory pathway is required for RANK-mediated osteoclastogenesis, and signals downstream through the protein kinase Syk (96, 97), which activates phospholipase Cg (PLCg) and the BTK and Tec kinases (98), eventually leading to calcium-mediated activation of NFATc1/NFAT2 (95). OPG functions as a soluble decoy-like factor for RANKL, and thus as a negative regulator of RANK signaling, and is capable of inhibiting osteoclastogenesis *in vitro*, and of inducing osteoporosis when transgenically overexpressed in mice (47). Furthermore, OPG-deficient mice are described as osteoporotic, with excessive numbers of OCs (99). OPG has further been shown to inhibit mature OC function *in vitro* (100). In addition to OPG, other negative regulatory mechanisms of RANK signaling have been described that inhibit osteoclastogenesis. For instance, though T cells can express RANKL, there is a negative correlation between T lymphocyte activation and signaling through RANK on OC precursors, as T cell-derived IFN-g drives proteasomal degradation of TRAF6 (42). In this way, a productive immune response is prevented from having an overlapping, deleterious effect on bone in the surrounding environment. TRAF6 activates downstream signaling via non-degradative ubiquitination (101). It has been demonstrated that TRAF6-mediated RANK signaling in pre-OCs is negatively regulated de-ubiquitinase CYLD, and that CYLD-deficient mice exhibit osteoporosis due to increased OC activity (36). Another regulatory mechanism involves negative feedback via by RANK-mediated upregulation of IFN-b, which mediates a feedback mechanism that blocks further c-fos-dependent activity (102). In support, it is reported that c-fos-deficient OC precursors exhibit deficient RANKL-mediated IFN-b production, and that mice deficient for the IFNa/b receptor (IFNAR1) exhibit osteoporosis characterized by an increase in OCs (102). A more recently characterized negative regulatory mechanism of RANK signaling in OC precursor cells involves the formation of a TRAF3-containing complex on the RANK intracellular domain that inhibits both canonical and non-canonical NF-κB pathways (43, 103). The physiologic relevance of this complex is demonstrated by TRAF3 conditionally deficient mice, which exhibit mild osteoporosis (43). Finally, an example of negative regulation of RANK-mediated ITAM activation has recently been described, which involves semaphorin 3A (Sema3A) interaction with neuropilin-1, and is supported by the finding that Sema3a-deficient mice are osteopenic (104). These data highlight the multiple levels of control that bone requires for proper homeostatic function, and suggest the potential that RANKL signaling has as a therapeutic target in treating bone-related ailments. In fact, denosumab, an anti-RANKL antibody is now in clinics for use in treating osteoporosis and shows promise for treating additional OC-related conditions (105).

## Inherited Bone Diseases

Though typically rare, various heritable mutations linked to the bone pathologies have been identified in the genes encoding the RANKL–RANK–OPG system. Familial expansile osteolysis (FEO) and Paget’s disease are rare autosomal dominant conditions characterized by enhanced bone remodeling and osteolytic lesions present in the long bones. Short in-frame duplications in exon 1 of the gene encoding RANK have been linked to FEO and Paget’s disease of the bone (PDB) (106). It has been shown that these mutations disrupt function of the RANK signal peptide and result in constitutive RANK activity (106). Expansile skeletal hyperphosphatasia (ESH) is a genetic disorder characterized by early onset deafness, premature loss of teeth, progressive hyperostotic widening of long bones causing painful phalanges in the hands, accelerated bone remodeling, and episodic hypercalcemia. While ESH is distinguished phenotypically from FEO by the presence of hypercalcemia and the absence of large osteolytic lesions with cortical thinning in major long bones, it appears also to occur from an activating mutation – in this case a 15-bp tandem repeat – in the region encoding the RANK signal peptide (107). Multiple additional mutations have been characterized in the gene encoding RANK that result in varied forms of osteopetrosis, but despite early onset, it has been shown in some cases that disease can be cured by hematopoietic stem cell transplant even when carried out in late infancy (108). Juvenile Paget’s disease is a rare autosomal recessive bone disease in which children are normal at birth, but then experience rapidly remodeling woven bone, osteopenia, fractures, and progressive skeletal deformity. Genetic analysis determined that this disorder is the result of an inactivating mutation in the gene encoding OPG, and that serum levels of OPG are undetectable in affected individuals (109). Cherubism is a rare autosomal dominant disease of the lower jaw characterized by excessive OC-mediated bone resorption and associated with mutations in the gene Sh3bp2 (110). While the mechanism(s) disease onset was not initially understood, more recent work has demonstrated that the mutations appear to impact regions of scaffolding protein encoded by Sh3bp2 that coordinate signals converging from RANK and M-CSFR to activate Syk, PLCg2, and Vav (111). These alterations in signaling complexes lead to increased TNF-a expression and augmented OC activity (111). Together these genetic cases confirm the critical role of the RANKL–RANK–OPG system in human bone development and function.

## Acquired Bone Pathologies

Much more common than bone pathologies caused directly by genetic lesions to the RANKL–RANK–OPG systems are bone-related pathologies that arise later due to environmental factors, homeostatic dysregulation, hormonal changes, or other disease sequelae. The most common of these is post-menopausal osteoporosis, a skeletal disorder characterized by weakening of the bones and predisposition to fracture due to bone loss caused by an imbalance in OC activity versus new bone formation (112, 113). Osteoporosis is associated with hormonal changes, such as decreased estrogen levels in post-menopausal women, and has been linked to increased RANKL levels on bone marrow cells of women exhibiting osteoporosis (113, 114). Similarly, patients receiving hormone ablation therapy for breast cancer (estrogen suppression) or prostate cancer (chemical or surgical castration for testosterone suppression) may also suffer osteoporotic bone loss due to increased RANKL expression (112, 113). These findings correlate with the osteoporotic phenotype observed in the OPG-deficient mouse model, in which RANKL–RANK interactions are enhanced due to the absence of the OPG-mediated inhibition (99). Cancer is another area where the RANKL–RANK–OPG system may affect bone health. In patients with bone metastases, skeletal complications caused by increased OC activity may result in pathological fractures, spinal cord compression, and the need for radiotherapy to the bone or orthopedic surgery [collectively known as skeletal-related events (SREs)] (115). Increased bone turnover may even enhance tumor growth in bone by facilitating the early establishment, as well as later progression, of bone metastases (116). Cancer metastases to bone result from engagement by tumor cells with non-malignant resident cells of the bone microenvironment, including OCs, stromal cells, and vascular cells, through cell–cell, paracrine, and/or endocrine interactions. The role of RANKL–RANK–OPG in metastasis can be divided into its contribution to enhanced osteolysis and to its effects on promoting metastasis. In the former case, some examples and mechanisms have been described. For instance, bone pain and excessive OC activity are the primary complication for multiple myeloma patients, with increased levels of RANKL often found in bone stromal cells (117). With respect to mechanisms of tumor-driven increases in RANK activity, one study showed tumor cell expression of metalloproteases ADAMTS1 and MMP1, factors associated with increased risk of metastasis in breast cancer, alter secretion of epidermal growth-like factors in a manner that suppresses OPG expression by resident OBs (118). In another study, it was shown that prostate cancer cells expressing a soluble form of RANKL could directly induce osteoclastogenesis from precursor cells in the absence of stromal accessory cells (119). With respect to promoting metastasis, interest in a potential role for RANKL–RANK was triggered by observations relating to its role in epithelial organogenesis, specifically mammary stem cell development, which could be envisioned as contributing to carcinogenic events (65, 115, 120). During tumor formation, RANKL is found to increase proliferation and survival of both normal and pre-neoplastic breast in addition to expansion of mammary stem/progenitor cells (65, 120). Progesterone and prolactin, which have been implicated in mammary tumorigenesis, both trigger RANKL expression in the mammary gland (121). In one study using a hormone-triggered mammary tumor model in mice, specific deletion of RANK in mammary epithelial cells significantly delays tumor onset (65, 121). Further, RANKL treatment is shown to protect mammary epithelial cells from γ-irradiation-induced cell death, one indicator of malignancy (65, 121). In a complementary study, transgenic mammary gland overexpression of RANK was shown to exacerbate medroxyprogesterone acetate-induced mammary tumor formation, and that systemic RANKL blockade resulted in a 90% reduction in hormone-induced mammary tumor onset (120, 121). With respect to showing the contribution of local differentiation factors, in addition to chemotactic factors, to metastasis, it was shown that RANKL stimulation directly triggered metastasis of melanoma cell lines and breast cancer in patients in a manner that is independent of pro-osteoclastic activity (122). A study investigating sources of RANKL outside of bone that may trigger metastasis showed that pulmonary metastasis of breast cancer may be driven by RANKL expressed on infiltrating Tregs, implicating the role of inflammatory factors in RANKL-driven metastasis (123). Finally, important work to determine the key molecular pathways downstream of RANK signaling in metastatic tumor cells showed a correlation between metastatic potential and RANK-induced IKK-a activation (124).

## Immunity and Osteoimmunology

**Figure 2:**
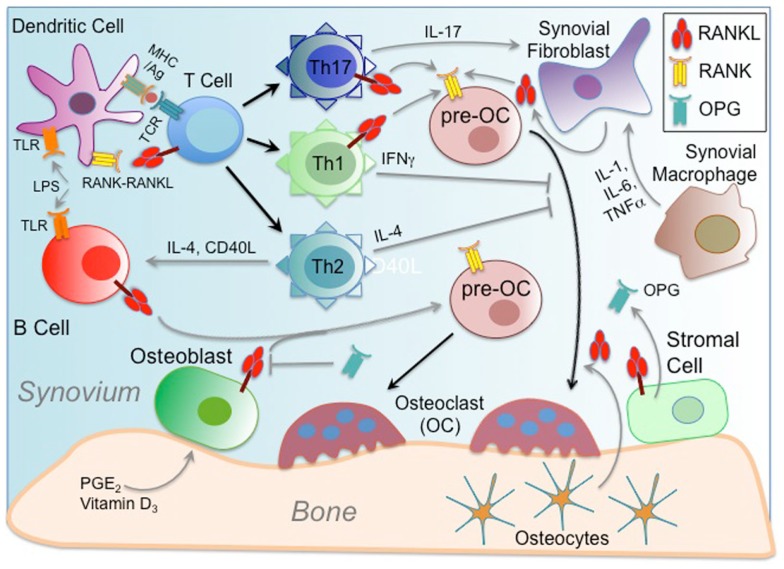
**Osteoimmunology and RANKL–RANK–OPG**. Osteoimmunology involves cross-regulation between cells of the bone and immune systems, and in some cases in the source of pathogenic conditions like rheumatoid arthritis (RA). The interface between the synovium and bone joints is where RA occurs, and where many cellular interactions typical of osteoimmunity have been characterized. The unifying characteristic of many of these cellular interactions is often the interplay between sources of RANKL and RANK-expressing cells. Secondarily, there are factors secreted or provided through cell contact that promote RANKL and/or RANK expression. The net effect of osteoimmune interactions is largely tallied according to increased (or regulation of) bone loss due to enhanced RANKL-mediated osteoclast (OC) differentiation from pre-OCs. In addition to the usual sources of RANKL available to pre-OCs from bone-associated cells including bone stromal cells, osteoblasts (OBs), and osteocytes, an inflammatory environment provides other sources. B cells activated by TLR ligands, such as LPS, and expanded by T cell help induce RANKL expression. T cells, which are activated by dendritic cells (DCs) through MHC/Antigen (Ag)–TCR interactions, can also express RANKL, which can both act on pre-OCs, but can also act on DCs to promote their survival and to prolong T–DC interactions. DC interactions with helper T cells influence their differentiation into subsets such as Th1, Th2, and Th17. Th1 and Th2 cell elaboration of IFNγ and IL-4, respectively, exhibit modulating effects on RANK-mediated osteoclastogenesis. However, IL-17 produced by Th17 cells can act to induce RANKL, especially by synovial fibroblasts under inflammatory conditions. Synovial macrophages may also enhance fibroblast expression of RANKL through secretion of inflammatory cytokines like IL-1, IL-6, and TNF-α. At the same time, mitigation of potentially deleterious effects of osteoimmune interactions may be provided by secretion of OPG, which attenuates the potency of available RANKL.

The RANKL–RANK–OPG system was initially discovered through multiple independent efforts, some interested in discovering new genes relevant to bone biology, and others initially focused on the immune system. As such, while initial efforts to characterize the role of RANKL–RANK–OPG in controlling osteoclastogenesis were ongoing, parallel efforts were underway showing that RANKL provided by T cells can significantly enhance immunity by promoting the survival and function of DCs, the most potent professional antigen-presenting cells, in the context of an immune response (4, 125, 126). Emerging understanding that key cellular regulators of the immune and bone systems were responsive to the same cytokine systems and derived from common progenitors (127) was one of the key impetuses in developing a new field of study, osteoimmunology, which seeks to examine the interactions between the bone and immune systems. Studies of RANK intracellular signaling pathways and regulatory mechanisms have further demonstrated the extent to which bone and immune cells overlap in these areas. Osteoimmunologic mechanisms are relevant to diseases including RA, periodontal disease, osteoporosis, osteoarthritis, multiple myeloma, and metastatic bone tumors, all of which are associated with bone breakdown (128). The most prominently studied example of the pathologic relationship between bone and immune cells is RA, but many RANKL–RANK–OPG-driven mechanisms of pathologic bone–immune cell interaction are common between different diseases (129) (depicted in Figure 2). RA is an autoimmune disease that is characterized by inflammation of the synovial joints, leading to severe structural damage including bone destruction. RANKL is highly expressed in the synovium of RA patients and is largely responsible for RA-related bone destruction (129). The source of pathogenic RANKL in RA synovium is still debated, as T cells may express high levels, but the osteoclastogenic action of T cells can be counteracted by IFN-g production (42). Instead, it appears that synovial fibroblasts are the primary RANKL source in RA (42). It has further been determined that Th17 helper T cells are responsible for inducing RANKL expression on synovial fibroblasts via expression of IL-17 as well as IL-1, TNF-a, and IL-6 (129). At the same time, a recent fate mapping study showed that more a potently osteoclastogenic version of pathogenic Th17 cells are those that were previously Foxp3-expressing Tregs, but that converted phenotypes, and gained RANKL expression in response to synovial fibroblast-derived IL-6 (130). Another study suggests that OC differentiation activity may not be the only function of RANKL in bone–immune cell interactions. Multi-photon microscopy was employed to perform intravital imaging of bone tissue in the context of RANKL-mediated OC activation, and it was showed that RANKL-expressing Th17 cells were able to stimulate mature but non-resorptive OCs to begin resorbing bone, suggesting that Th17-mediated bone pathology may not necessarily generate need OCs, but simply increase activity of mature resident OCs (131). In another model system, a recent study showed that RANKL expression by B cells drive OC formation in an ovariectomy (ovx) model of osteoporosis, suggesting that B cells should be examined more closely in bone–immune cell interactions (132). For normal bone remodeling, OB or bone stromal cells have long been considered the major sources of RANKL, but recent work employing cell-specific RANKL deletion suggests that osteocytes are in fact the critical providers of RANKL to OC precursors (86, 133). It is therefore clear that the cellular source of RANKL is critical to the context in which it is acting, and whether it primarily affects bone or immune cells. In addition to activating the immune system through RANK signaling on DCs, RANKL is conversely important for inducing immune tolerance by promoting Treg differentiation in certain autoimmune contexts. RANKL is required for Tregs that prevent cytotoxic destruction of pancreatic beta islet cells in a mouse type-1 diabetes model (134), as well as for Treg-mediated control of a colitis model (135). RANKL may promote peripheral immune tolerance. For instance, it has been reported that RANKL-expressing keratinocytes in inflamed skin trigger epidermal DCs to induce a Treg phenotype in infiltrating T cells (136). At the same time, another autoimmune disorder has recently been revealed, at a clinical level, to harbor a deleterious role for RANKL. High serum levels of soluble RANKL apparently correlate with risk for development of type-2 diabetes mellitus (T2DM). A recent study has identified a mechanism underlying this risk factor as hepatic insulin resistance induced by the RANK–NF-κB signaling pathway (137). Blockade of hepatic RANKL was able to ameliorate disease and lower plasma glucose levels, highlighting a potential strategy for treating T2DM (137). Finally, an example of modulation of RANKL function at the level of central immune tolerance may have important clinical implications. It was recently shown that mTEC inhibition via blockade of RANKL may represent a viable approach to boosting anti-tumor T cell responses by temporarily disrupting thymic negative selection to TSAs expressed by tumors (138). Together these examples show that RANKL–RANK either activating or suppressive to an immune response depending on the context, and that while important roles for other cytokines have been identified [e.g., IL-1, IL-6, IL-17, IL-23, TNF-a, and TGF-b (129)], RANKL remains the most critical means of communication between cells of the osteoimmunologic network.

## Concluding Remarks and Future Directions

It is difficult to overstate the importance of the discovery of the RANKL–RANK–OPG system with respect to understanding how bone homeostasis is controlled. The hundreds of studies it spurred have uncovered a much more vast biological network of regulation involving RANKL–RANK in and across other organ systems, and have as an additional benefit, demonstrated previously unknown ways in which organ systems interact and cross-regulate at a molecular level. Now that RANKL–RANK has been successfully harnessed for purposes of therapeutic treatments of osteoporosis, bone loss, and bone metastasis, it will be important to answer additional questions – specifically with respect to how RANK signaling is modulated, and how and on what cells RANKL is physiologically expressed under normal versus disease conditions – if therapeutic RANKL–RANK targeting is to be refined and potentially applied to additional disease conditions.

## Conflict of Interest Statement

The authors declare that the research was conducted in the absence of any commercial or financial relationships that could be construed as a potential conflict of interest.
